# Supplementary material for: Spatial and single‐nuclei transcriptomics reveals idiosyncratic and generic patterns in papillary and anaplastic thyroid cancers
Source: Mol Oncol. 2026 Aug 11:10.1002/1878-0261.70289. Online ahead of print. doi: 10.1002/1878-0261.70289 (PMC13459077; doi:10.1002/1878-0261.70289)
Supplement: Supplementary file 1 [file MOL2-9999-0-s002.pdf]

Supplemental material

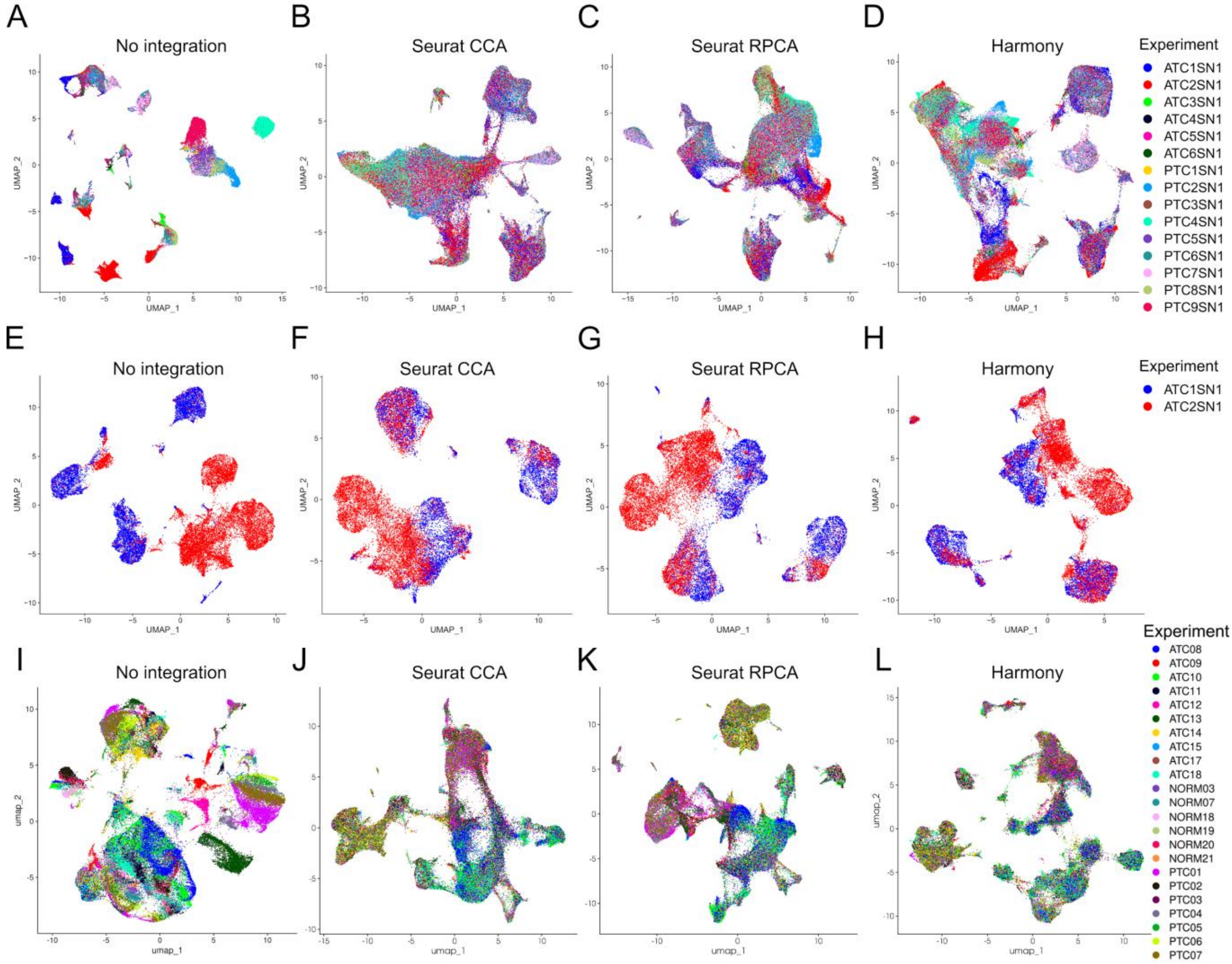

Supplemental Figure 1. Three integration methods conceal inter-sample variation in three datasets. (A) UMAP (Uniform manifold approximation and projection) of 9 PTC (Papillary thyroid cancer) and 6 ATC (Anaplastic thyroid cancer) samples without integration, **(B)** with Seurat's canonical correlation analysis integration, **(C)** with Seurat's reciprocal principal component analysis integration, **(D)** with Harmony integration. **(E)** UMAP of samples ATC1 and ATC2 without integration, **(F)** with Seurat's canonical correlation analysis integration, **(G)** with Seurat's reciprocal principal component analysis integration, **(H)** with Harmony integration. **(I)** UMAP of samples from Lu et al. with 6 normal, 7 PTC and 10 ATC samples without integration, **(J)** with Seurat's canonical correlation analysis integration, **(K)** with Seurat's reciprocal principal component analysis integration, **(L)** with Harmony integration.

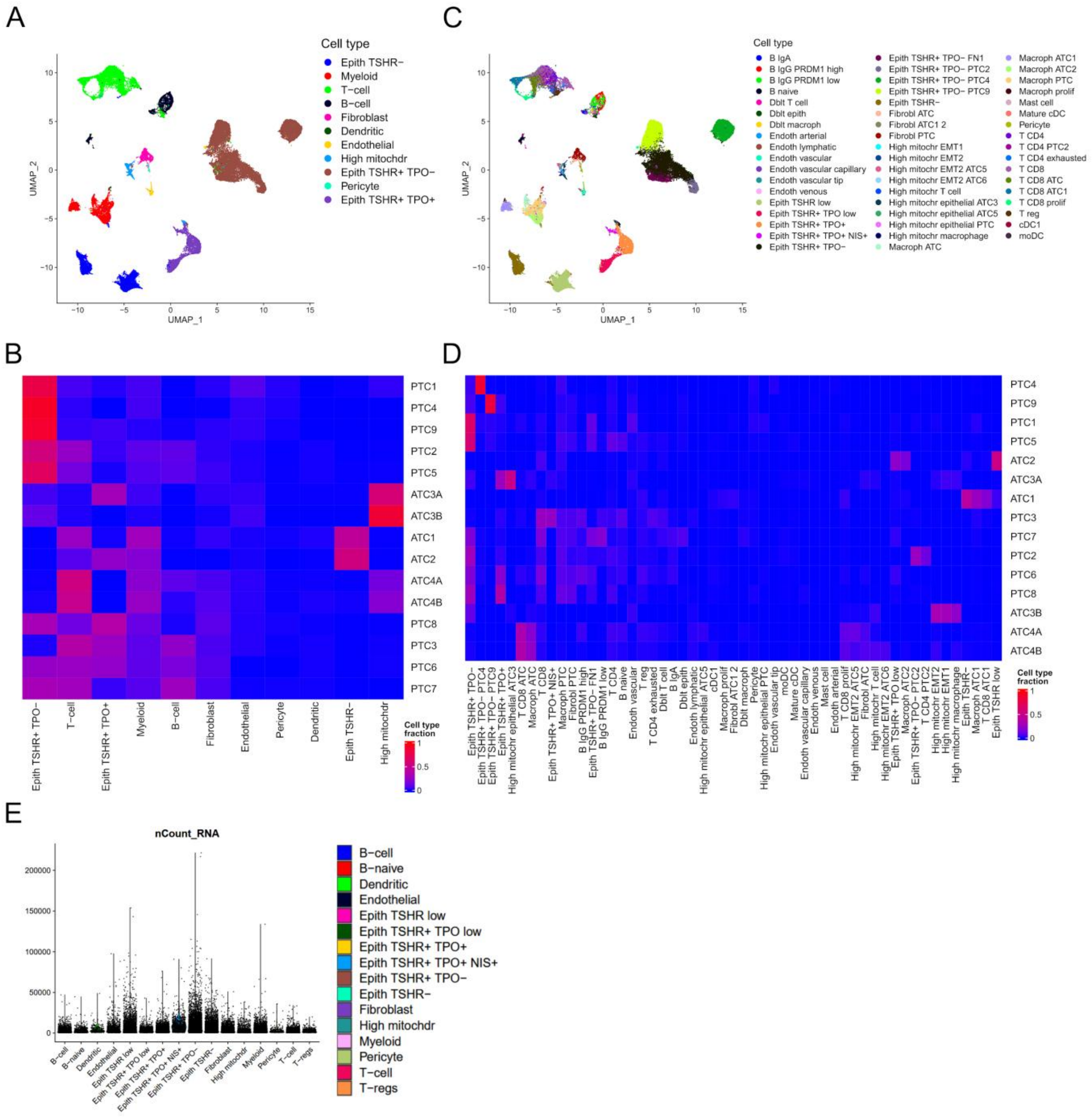

Supplemental Figure 2. Coarse and fine grained cell types and cell states compositions. (A) Low resolution annotation of 9 PTC (Papillary thyroid cancer) and 6 ATC (Anaplastic thyroid cancer) samples. **(B)** Low resolution annotation composition of 9 PTC and 6 ATC samples. **(C)** High resolution annotation of 9 PTC and 6 ATC samples. **(D)** High resolution annotation composition of 9 PTC and 6 ATC samples. **(E)** Number of unique molecular identifiers across cell types for 9 PTC and 6 ATC samples. Sequencing depth alone does not explain the loss of thyroid markers.

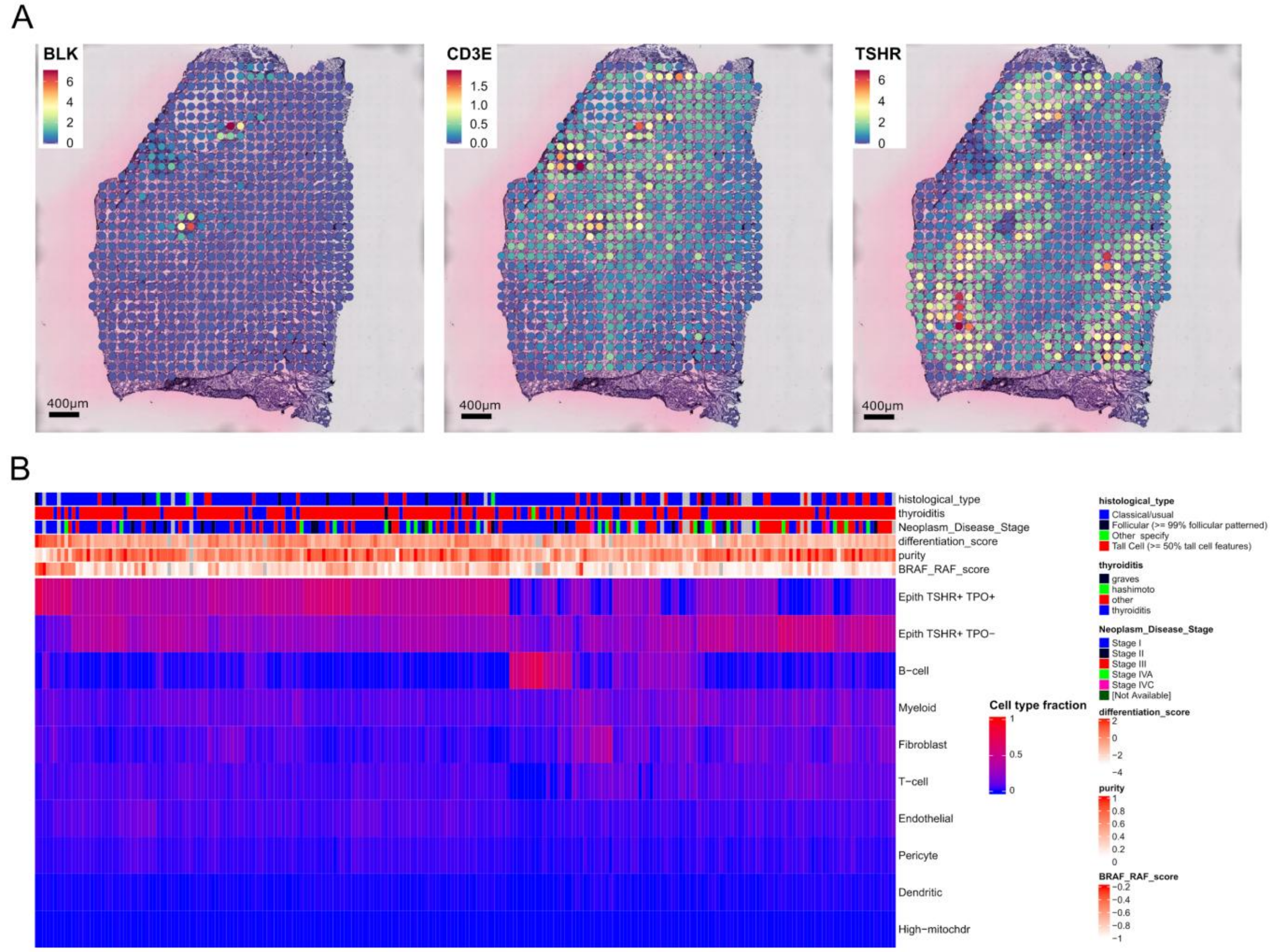

Supplemental Figure 3. Immune cell abundance in *BRAF^{V600E}* Papillary thyroid cancer. (A) Expression level of immune and epithelial markers in PTC6S1. Scale bars = 400µm. **(B)** Cibersortx-inferred cell type composition of 234 *BRAF^{V600E}* mutated samples from The Cancer Genome Atlas.

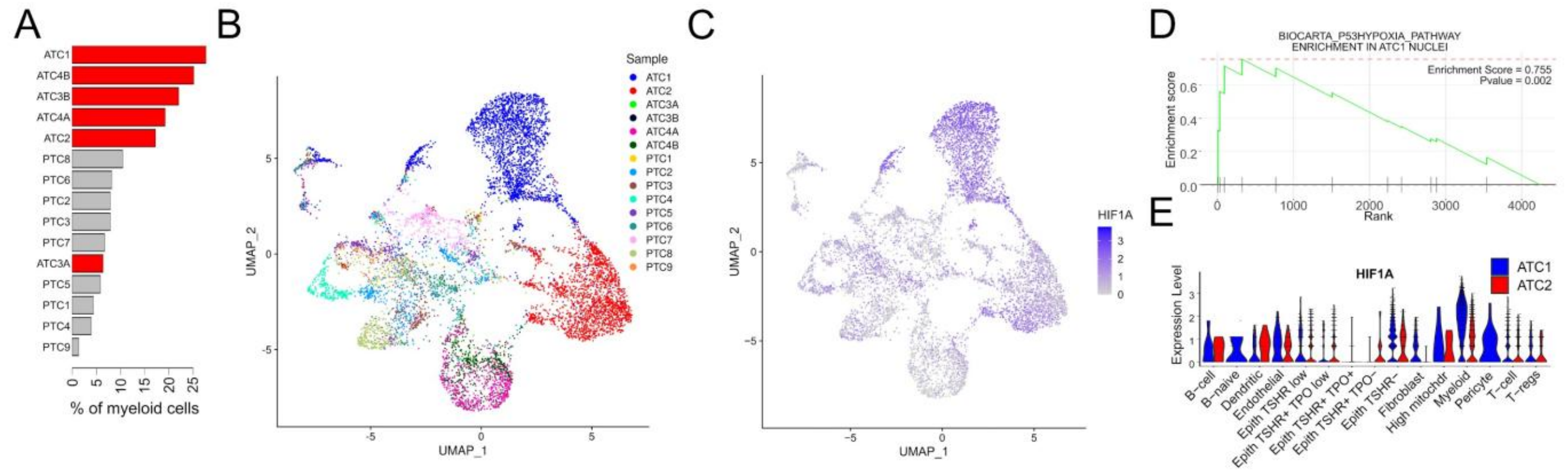

Supplemental Figure 4. An idiosyncratic myeloid cell state is associated with hypoxia in an ATC. **(A)** Fraction of myeloid cells in snRNA-seq (single nuclei RNA sequencing) samples. Red depicts ATC (Anaplastic thyroid cancer) (N = 15). **(B)** UMAP (Uniform manifold approximation and projection) of myeloid cells with sample annotation. Cells from ATC1 and ATC2 form distinct clusters. **(C)** Expression of *HIF1A* gene in myeloid cells. It is increased in ATC1. **(D)** Enrichment for hypoxia pathway in ATC1 myeloid nuclei (p-value = 0.002). **(E)** Expression level of gene *HIF1A* across cell types for samples ATC1 and ATC2. *HIF1A* is increased in all cell types present in ATC1, pointing to systemic hypoxia.

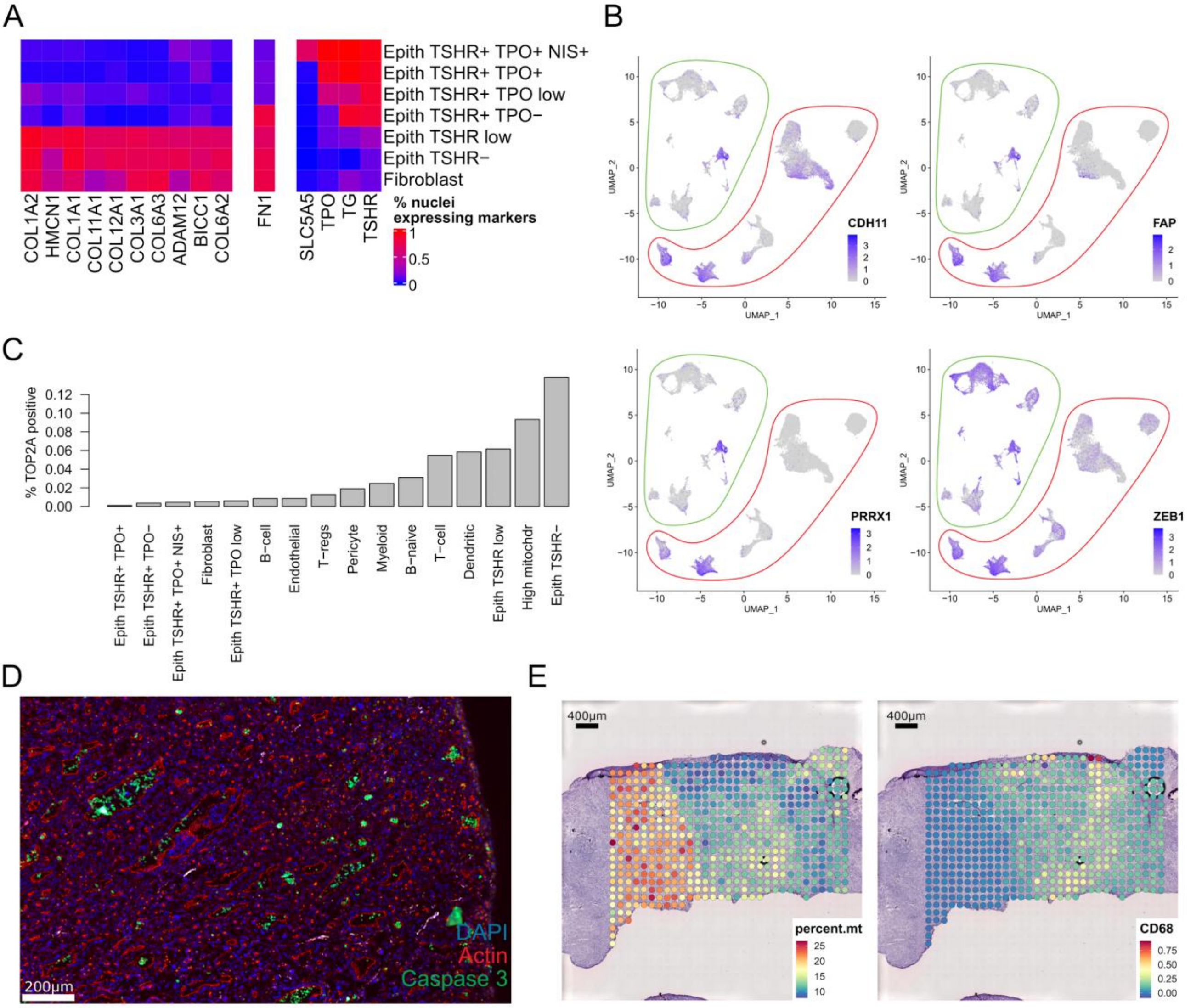

Supplemental Figure 5. *TSHR$^{low/-}$* cells, epithelial-mesenchymal transition, and cell death. (A) Expression level of mesenchymal markers in 9 PTC (Papillary thyroid cancer) and 6 ATC (Anaplastic thyroid cancer) samples. The microenvironment is highlighted in green and nuclei of epithelial origin in red. **(B)** Fraction of cells expressing mesenchymal and thyroid differentiation markers in the fibroblast and epithelial subsets. **(C)** Fraction of *TOP2A* positive nuclei per cell type. **(D)** Immunofluorescence staining of ATC3 showing cells positive for the activated form of Caspase3 (N=1). Apoptotic cells stained for Caspase3 are primarily located in the follicular lumens. Scale bar = 200µm. **(E)** Expression level of mitochondrial genes and *CD68* in sample ATC3BS1. **(F)** Expression level of *TG* for all subsets of the medium resolution annotation in 9 PTC and 6 ATC samples. It demonstrates that apparent TG expression in fibroblasts panel B is likely a non cell type-specific effect of ambient TG RNA contamination. Scale bars = 400µm.

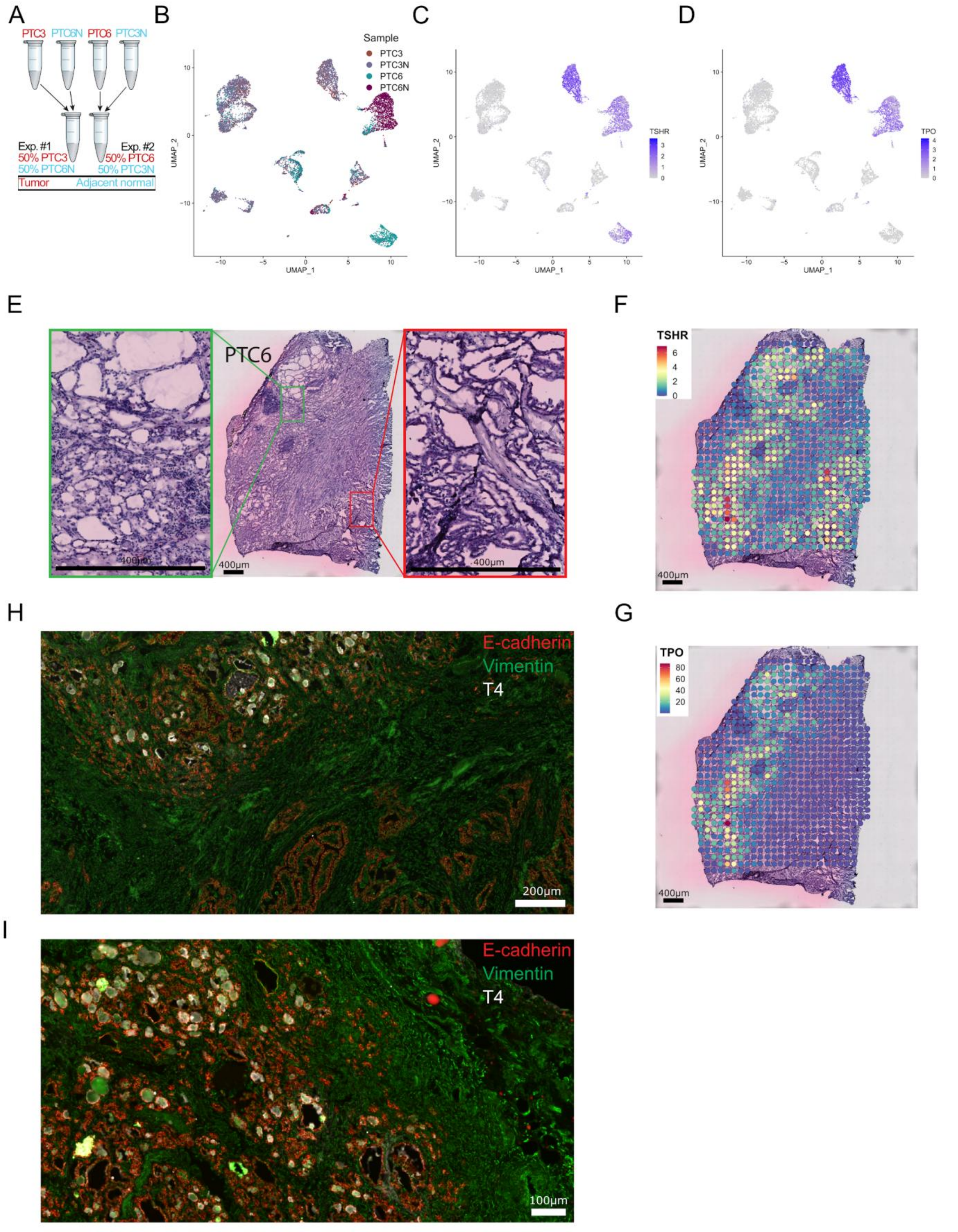

Supplemental Figure 6. A cell mixing experiment resolves the status of TPO⁺ cells. Tumor slices could contain adjacent normal nuclei, raising doubts about the status of TPO⁺ cells: are they surprisingly well differentiated cancer cells or normal contaminants? As our original study design did not explicitly include normal samples, we performed additional experiments mixing tumor and adjacent healthy

tissues to settle this question. **(A)** Schematic representation of the nuclei mixing experiment for samples PTC3, PTC6 and their adjacent normal tissue. **(B)** UMAP (Uniform manifold approximation and projection) showing the sample of origin of each nuclei as assigned by Vireo for samples PTC3, PTC6 and their adjacent normal tissue. **(C, D)** Expression level of *TSHR* (**C**) and *TPO* (**D**) in PTC3, PTC6 and their adjacent normal tissue. Epithelial nuclei from PTC3, PTC3N and PTC6N express *TPO* and *TSHR*, while those from PTC6 only express *TSHR*. This is corroborated by spatial data. **(E)** H&E image of PTC6 with zoom on the hormone-producing area (left) and the PTC-like area (right). Scale bars = 400µm. **(F, G)** Expression level of *TSHR* (**F**) and *TPO* (**G**) in PTC6. Scale bar = 400µm. **(H)** Immunofluorescence staining of PTC6 at the intersection between hormone-producing and PTC-like areas (N=1). T4 staining (white) is consistent with the *TPO* expression pattern of panel **G**. Scale bar = 200µm. **(I)** Immunofluorescence staining of PTC6 normal adjacent sample (N=1). Scale bar = 100µm.

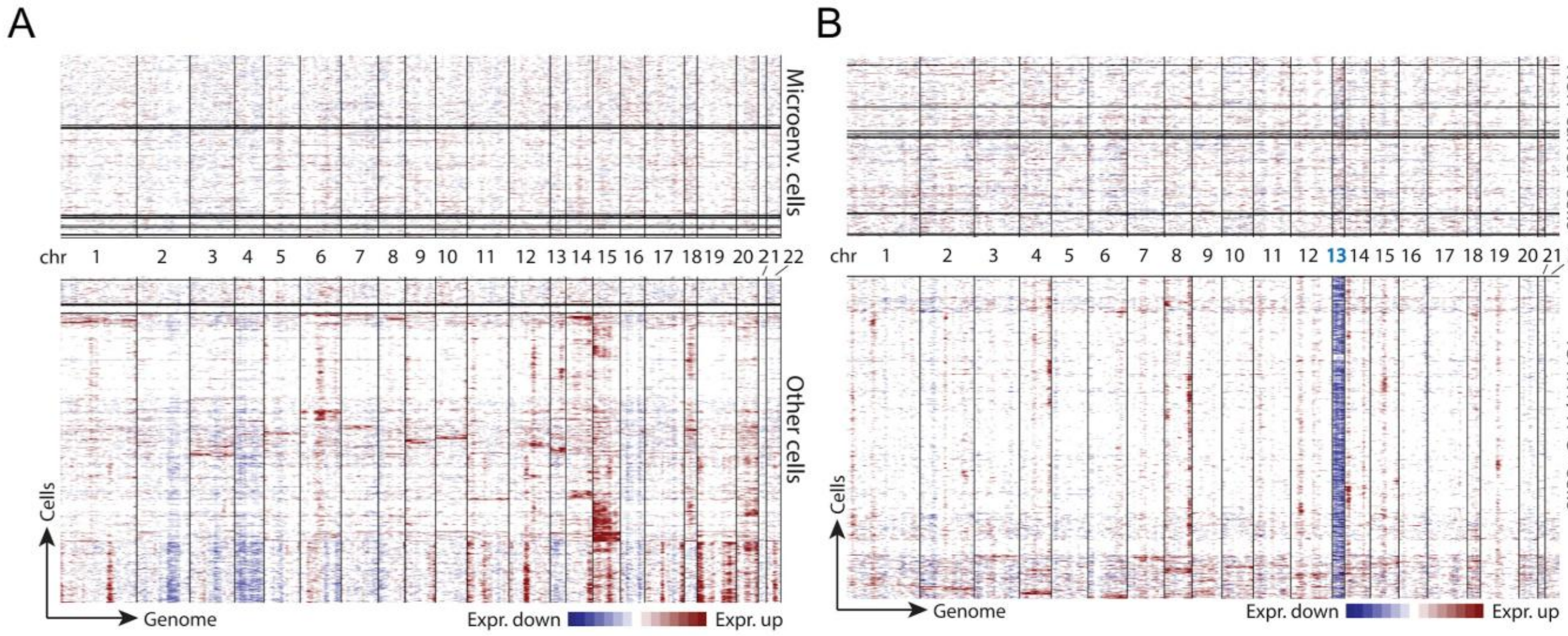

Supplemental Figure 7. DNA copy number profiles of *TSHR*$^{low/-}$ cells. (A) DNA copy number alterations inferred from snRNA-seq (single nuclei RNA sequencing) by infercnv in sample ATC1. **(B)** DNA copy number alterations inferred from snRNA-seq by infercnv in sample PTC4.

A

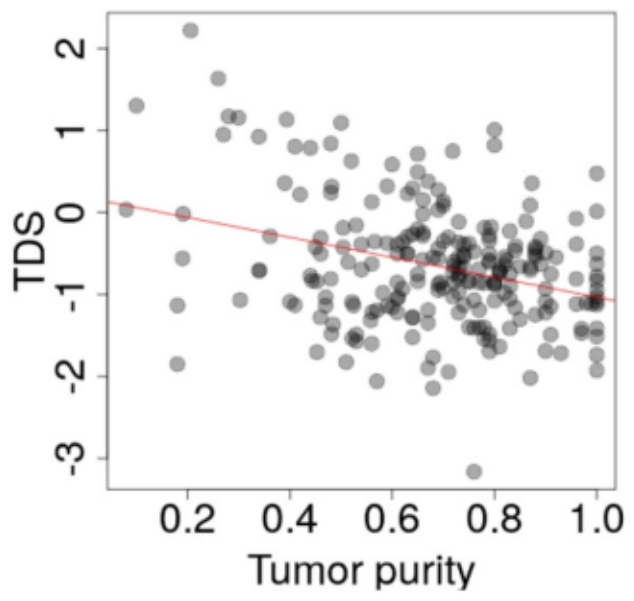

Supplemental Figure 8. Correlation between TDS and purity in the TCGA cohort. (A) Scatterplot of the thyroid differentiation score (TDS) vs. tumor purity in the BRAF-mutated PTC (Papillary thyroid cancer) samples of the TCGA (The Cancer Genome Atlas) cohort. Spearman's correlation between TDS and purity is $\rho = 0.32$, $p < 2x10^{-16}$.
